# Salivary histatin 3 inhibits heat shock cognate protein 70-mediated inflammatory cytokine production through toll-like receptors in human gingival fibroblasts

**DOI:** 10.1186/1476-9255-11-4

**Published:** 2014-02-04

**Authors:** Yasuhiro Imamura, Pao-Li Wang

**Affiliations:** 1 Department of Pharmacology, Matsumoto Dental University, Shiojiri, Nagano, Japan; 2 Department of Dental Education Innovation, Osaka Dental University, Hirakata, Osaka, Japan

**Keywords:** Histatin, Inflammatory cytokine, Toll-like receptor, HSC70

## Abstract

**Background:**

Salivary histatins are bioactive peptides related to the innate immune system associated with antimicrobial activities. However, very little is known about the physiological and biological functions of histatins against host cells or their role in oral cell inflammation. Histatin 3 binds to heat shock cognate protein 70 (HSC70, a constitutively expressed heat shock protein (HSP)). It is unclear whether HSC70 is involved in the inflammatory response in oral cells. Injured oral cells release some intracellular proteins including HSC70. It is possible that released HSC70 induces toll-like receptor (TLR) activation, just as extracellular HSP70 (a stress inducible HSP) does, and that histatin 3 affects this process. Therefore, we tested the hypothesis that HSC70 activates TLR signaling and histatin 3 inhibits this activation and inflammatory cytokine production.

**Methods:**

A nuclear factor (NF)-κB-dependent luciferase reporter plasmid was transfected into HEK293 cells stably expressing TLR2 with coreceptor CD14 (293-TLR2/CD14 cells) or stably expressing TLR4 with CD14 and the accessory molecule MD2 (293-TLR4/MD2-CD14 cells). The cells were stimulated with HSC70 in the presence or absence of histatin 3, and examined using luciferase assays. We also stimulated human gingival fibroblasts (HGFs) with HSC70 with or without histatin 3. Then, we analyzed the levels of inflammatory cytokines (interleukin (IL)-6 and IL-8) in the culture media. Cell proteins were analyzed using enzyme-linked immunosorbent assay and Western blotting with antibodies of mitogen-activated protein kinases and NF-κB inhibitor IκB-α, respectively. Histatin 3-bound form of HSC70 was analyzed using limited V8 protease proteolysis.

**Results:**

HSC70 induced NF-κB activation in a dose-dependent manner in 293-TLR2/CD14 and 293-TLR4/MD2-CD14 cells, and histatin 3 inhibited this process and when histatin 3 binding to HSC70 was precluded by 15-deoxyspergualin, which augmented NF-κB-triggered activation. In HGFs, histatin 3 also inhibited HSC70-induced inflammatory cytokine production, extracellular signal-regulated protein kinase phosphorylation, and degradation of IκB-α. Moreover, HSC70 in the presence of histatin 3 was relatively resistant to digestion by V8 protease compared with HSC70 in the presence of control peptide.

**Conclusions:**

Histatin 3 may be an inhibitor of HSC70-triggered activation of TLR signaling and inflammatory cytokine production and may be involved in inflammation processes noted in oral cells.

## Background

Histatins constitutively secreted by the salivary glands are associated with innate immunity processes in the oral cavity. These peptides have antimicrobial properties and protect oral tissues from pathogens [1]. The histatin family comprises 12 histidine-rich cationic peptides found in healthy adults at concentrations of 50–425 μg/ml, corresponding to approximately 10% of total protein in saliva [2-4]. Histatins 1 and 3 are full-length peptides of 38 and 32 amino acid residues, respectively; other characterized members of the histatin family are proteolytic products formed during secretion [2,5]. Histatins 3 and 5 are the heat shock protein (HSP)-binding proteins that are most abundant in saliva and are active against *Candida albicans* and *Porphyromonas gingivalis* (the pathogen of periodontitis) [6-10]. In addition, histatins 3 and 5 are also involved in proliferation of human gingival fibroblasts (HGFs) and rabbit costal chondrocytes, respectively [7,11].

HSPs are induced by a wide variety of stresses in prokaryotic and eukaryotic cells, including environmental, pathological, and physiological stimuli [12]. HSPs function as ATPase activity-dependent molecular chaperones that assist in the correct folding of proteins, the assembly of various protein complexes, transport of proteins across membranes into organelles, and the degradation of proteins by the lysosome [13-15]. Heat shock cognate protein 70 (HSC70), an HSP family member, is a cytosolic protein that is abundantly, constitutively, and ubiquitously expressed in most cells [16]. HSC70 consists of an ATPase domain (amino acid residues 1–384), a substrate (peptides, including histatin 3)-binding domain (amino acid residues 385–543), and a lid domain (amino acid residues 544–646) [7,17]. Three-dimensional structure of the ATPase domain has been determined by X-ray crystallography; the structure of this domain is similar to that of hexokinase and actin [18,19].

Toll-like receptors (TLRs) have been identified as human homologs of *Drosophila* Toll receptors, which are involved in innate immunity [20,21]. TLRs recognize their respective pathogen-associated molecular patterns (PAMPs) and damage-associated molecular patterns (DAMPs, such as intracellular proteins released from damaged and necrotic cells) [22]. TLR2, a TLR family member, recognizes peptidoglycan (PGN, a major cell-wall component of gram-positive bacteria), lipopolysaccharide (LPS) from *P. gingivalis*, and HSP70 (a stress-induced HSP) [23-25]. TLR4 is another TLR family member, which recognizes LPS from the outer membranes of gram-negative bacteria and HSPs such as HSP70, HSP60, and Gp96 [25-28]. TLR2 and TLR4 require their interacting proteins for the recognition of some PAMPs and DAMPs. TLR2 can induce nuclear factor (NF)-κB activation in response to HSP70 stimulation, but only in the presence of CD14, a glycosylphosphatidylinositol-anchored protein [25]. In order for TLR4 to function satisfactorily as a receptor for LPS, both MD2 and CD14 must be coexpressed [29,30]. Once TLR2 and TLR4 recognize their respective ligands, the specific response initiated by these TLRs depends on the recruitment of adaptor proteins (e.g., myeloid differentiation primary response protein 88 or Toll-interleukin (IL)-1 receptor domain-containing adaptor protein). These adaptor proteins transmit signals that result in the activation of mitogen-activated protein kinases (MAPKs) and NF-κB and the induction of inflammatory cytokines [31].

It is not known whether HSC70 is involved in the inflammatory responses in oral cells, such as the production of inflammatory cytokines. It has been proposed that oral diseases accompanying damage or oral injuries cause the release of some intracellular proteins, including HSC70. If HSC70, like HSP70, functions as DAMPs, HSC70 could be a putative inducible factor in the inflammatory response in oral cells through TLRs. HGFs, which constitute the major cellular population of gingival tissue, express TLR2, TLR4, MD2, and CD14 [32-34]. In addition, histatin 3 in saliva may associate with the released HSC70. Therefore, we can infer that histatin 3 inhibits TLR-mediated HSC70 function, reducing the production of inflammatory cytokine in oral cells.

In this study, we identified HSC70 as a putative ligand of TLR2 and TLR4. We observed the inhibitory effect of histatin 3 on inflammatory cytokine production in HGFs and on NF-κB activation in HEK293 cells expressing TLR2 or TLR4 as a result of HSC70 stimulation. These findings represent our current knowledge of physiological functions of salivary proteins in oral cavity.

## Methods

### Cell cultures

The stable cell lines, 293-TLR4/MD2-CD14 (InvivoGen) and 293-TLR2/CD14 (InvivoGen) and HGFs were cultured in Dulbecco’s modified Eagle medium (DMEM; Sigma-Aldrich) with 10% fetal bovine serum (FBS), 100 units/ml of penicillin G, and 100 μg/ml of streptomycin at 37°C in 5% CO_2_ and 95% air in a humidified incubator. HGFs were collected from volunteers after obtaining appropriate informed consent. The Ethics Committee of Matsumoto Dental University approved the study protocol.

### Reagents

The following materials and antibodies were purchased: LPS from *Escherichia coli* 0128:B12 (Sigma-Aldrich); LPS from *P. gingivalis* (InvivoGen); recombinant human HSP70 (Stressgen; endotoxin activity, <0.05 endotoxin units (EU)/μg); recombinant bovine HSC70 (Stressgen; <0.05 EU/μg); recombinant bovine HSC70 ATPase fragment (Enzo Life Sciences; 0.1 EU/μg); V8 protease (Roche); 15-deoxyspergualin (DSG) (Spanidin® Inj.; Nippon Kayaku); mouse monoclonal anti-TLR4 (HTA125) and anti-TLR2 (TL2.1) antibodies (Hycult Biotechnology); mouse monoclonal anti-CD14 (MY4) antibody (Beckman Coulter); rabbit polyclonal anti-p44/42, anti-p38, anti-phosphorylated p38, anti-phosphorylated IκB-α, mouse monoclonal anti-phosphorylated p44/42 antibodies (Cell Signaling); mouse monoclonal anti-JUN-N-terminal protein kinase (JNK) (D-2), anti-phosphorylated JNK (G-7), anti-IκB-α (H-4) antibodies (Santa Cruz Biotechnology); and mouse monoclonal anti-β-actin antibody (Abcam). Endotoxin levels of all materials used were determined using a ToxinSensor chromogenic LAL endotoxin assay kit (GenScript), and the resultant level was low (~0.1 EU/μg). The LPS, bovine serum albumin (BSA), HSP70, HSC70, and the HSC70 ATPase fragment were heated at 95°C for 20 min.

### Peptides

Human histatins 3, 4, and 5 and P3a peptide (Biosynthesis Inc.) were chemically synthesized [7]. The control peptide, (Pro-Pro-Gly)_10_ · 9H_2_O, was purchased from Peptide Institute Inc.

### Transfection and luciferase assays

pIgκB-Luc (1 μg) and pRSV-β-gal (0.1 μg), as the standard plasmid, were mixed with TransIT-LT1 transfection reagents (Mirus) [35]. The mixtures were transfected into 3 × 10^5^ cells. One day after transfection, the cells were stimulated using LPSs from *E. coli* (20 ng/ml) or *P. gingivalis* (100 ng/ml). For 293-TLR4/MD2-CD14 cells, the incubation mixtures also contained 1.4 or 14 nM BSA, HSP70, or HSC70 or 14 nM heated HSP70, heated HSC70, or the HSC70 ATPase fragment (heated or unheated). For 293-TLR2/CD14 cells, the incubation mixtures contained 2.8 or 28 nM BSA, HSP70, or HSC70 or 28 nM heated HSP70, heated HSC70, or the HSC70 ATPase fragment (heated or unheated). Cells were harvested and lysed 6 h after stimulation. Luciferase and β-galactosidase activities in the lysates were measured as described previously [35]. The luciferase activities were compared after normalization against the standard (β-galactosidase activity). For the peptide experiments, the stimulant (20 ng/ml, *E. coli* LPS or 14 nM, HSP70 or HSC70 for 293-TLR4/MD2-CD14 cells; 100 ng/ml, *P. gingivalis* LPS or 28 nM, HSP70 or HSC70 for 293-TLR2/CD14 cells) and respective peptides (3 or 30 μM; stimulation with peptide alone, 30 μM) were mixed. For the DSG experiments, HSC70 (14 nM for 293-TLR4/MD2-CD14 cells and 28 nM for 293-TLR2/CD14 cells) and the peptides (30 μM) were mixed with DSG (10 μg/ml). All the above mixtures were placed on ice for 30 min before being added to transfected cells. The cells were then cultured for 6 h. Figures show representative examples of 3 identical experiments with essentially identical results.

### Western blotting

HGFs (1.2 × 10^5^) were cultured in DMEM containing 0.1%-0.5% FBS for 24 h. Cells were stimulated using 10 ng/ml LPS or 0.5 μg/ml HSC70 for the indicated time periods. For peptide experiments, 1.5 μg/ml HSC70 and 0.05 or 0.5 μM peptides (stimulation of peptide alone, 0.5 μM) were mixed and placed on ice for 15 min. The mixtures were added to low serum-cultured HGFs. After 30 min, the cells were lysed with radioimmunoprecipitation assay (RIPA) buffer (50 mM, Tris–HCl (pH 7.5); 5 mM, EDTA; 150 mM, NaCl; 1% NP-40; 0.1% sodium dodecyl sulfate (SDS); 0.5% sodium deoxycholate; 1 mM, phenylmethylsulfonyl fluoride; 10 μg/ml, leupeptin; 10 μg/ml, aprotinin; 5 mM, Na_3_VO_4_; 10 mM, NaF). Western blotting analyses were performed as previously described [35,36]. Figures of Western blotting are representative of 3 independent experiments with similar results. Relative band intensity was measured using the ImageJ software (http://imagej.nih.gov/ij/).

### Enzyme-linked immunosorbent assays (ELISAs)

HGFs (1 × 10^4^) were cultured with HSC70 (7 or 70 nM), heated HSC70 (70 nM), or the unheated or heated HSC70 ATPase fragment (70 nM) for 24 h. For antibody experiments, HGFs were cultured with 10 μg/ml anti-TLR4, anti-TLR2, or anti-CD14 antibodies for 1 h. LPSs (from *E. coli*, 10 ng/ml; from *P. gingivalis*, 5 ng/ml) and HSC70 (70 nM) were then added to the cells, and the cells were cultured for 24 h. For peptide experiments, HSC70 (70 nM) and peptides (0.15 μM and 1.5 μM) were mixed and placed on ice for 15 min. The mixtures were added to the cells and cultured for 24 h. The culture media from all the above experiments were collected, and levels of IL-6 and IL-8 were measured using CytoSet kits (Biosource). Figures show representative examples of three identical experiments with essentially identical results.

### Limited proteolysis with V8 protease

HSC70 (2.1 μM) and peptides (10.5 μM) in a buffer (50 mM, Tris-HCl (pH 8.0); 0.5 mM, DTT; 150 mM, NaCl) were placed on ice for 10 min. After the addition of V8 protease (75 or 750 ng), the mixtures were incubated at 30°C for 1.5 h. The reactions were terminated by the addition of 0.5 volume of 2× SDS sample buffer (140 mM, Tris–HCl (pH 7.0); 6% SDS; 10% mercaptoethanol; 22.4% glycerol; 0.02% bromphenol blue; and 2 mM, phenylmethylsulfonyl fluoride). The samples were heated at 100°C for 5 min, and the proteolytic fragments were resolved using 12% SDS-polyacrylamide gel electrophoresis (PAGE) gels, followed by staining with Coomassie Brilliant Blue. Figure is representative of 3 independent experiments with similar results.

### Statistical analysis

All quantitative data were statistically analyzed using either one-way analysis of variance (ANOVA) or two-way ANOVA using the StatMate software (ATMS). Differences were considered statistically significant at P < 0.05.

## Results

### NF-κB activation by stimulation with exogenous HSC70 through TLR2 and TLR4

**Figure 1:**
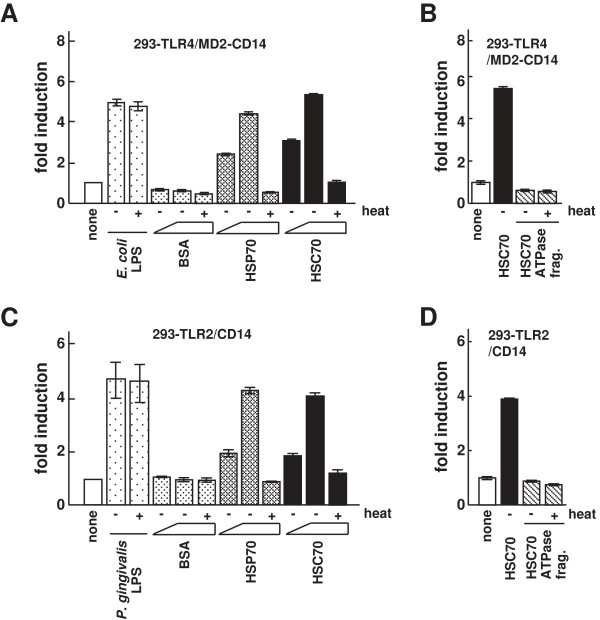
**The effect of HSC70 on NF-κB-dependent activation through TLR2 and TLR4. (A)** Luciferase assays for NF-κB-dependent activation by HSC70 through TLR4. 293-TLR4/MD2-CD14 cells were transfected with an NF-κB-dependent luciferase reporter plasmid (pIgκB-Luc). One day after transfection, cells were stimulated with heated and unheated *E. coli* LPS, BSA, HSP70, or HSC70 for 6 h. Cell lysates prepared from the stimulated cells were analyzed by luciferase assays. The values are shown as fold induction of the standardized luciferase activity over the unstimulated control (none). **(B)** Luciferase assays for NF-κB-dependent activation by the ATPase fragment of HSC70 in 293-TLR4/MD2-CD14 cells. The procedures were as in **(A)**, except that the cells were stimulated with the heated or unheated HSC70 ATPase fragment. **(C)** Luciferase assays for NF-κB-dependent activation by HSC70 through TLR2. The procedures were as in **(A)**, except that 293-TLR2/CD14 cells and *P. gingivalis* LPS were used. **(D)** Luciferase assays for NF-κB-dependent activation by the HSC70 ATPase fragment in 293-TLR2/CD14 cells. The procedures were as in (A and B). Bars represent the means and range of duplicate samples.

HSP70 stimulates inflammatory cytokine production (e.g., IL-6 and tumor necrosis factor-α) in monocytes and dendritic cells through TLR2 and TLR4 [25,37]. However, the activation of inflammatory cytokine production by HSC70, even though it belongs to the same HSP family as HSP70, is poorly understood. Therefore, we investigated whether cytokine production by HSC70 is mediated by TLRs and their interacting proteins such as HGFs, particularly in oral cells. Because HGFs express TLR2 and TLR4, it is necessary to perform systematic experiments investigating the dependence of HSC70 stimulation on TLR2 or TLR4. It has been reported that HSP70 stimulation induces NF-κB activation in 293T cells transiently expressing TLR4 and MD2, but not in the cells expressing TLR4 alone [38]. In contrast, till now there has been no evidence that HSC70 activates NF-κB in HEK293 cells expressing TLRs. Therefore, we examined NF-κB activation in the stable cell line 293-TLR4 (HEK293 cells constitutively expressing TLR4) transiently transfected with an MD2 expression vector and an NF-κB-dependent luciferase reporter plasmid. The results indicated that HSC70 stimulated NF-κB-dependent promoter activity through TLR4/MD2 signaling pathway. Moreover, when CD14 and MD2 were both expressed in 293-TLR4 cells, the promoter activation by HSC70 was more effective than that in 293-TLR4 cells transiently expressing MD2 alone (data not shown). We then examined whether this activation was dose-dependent. The stable cell line 293-TLR4/MD2-CD14 (HEK293 cells constitutively expressing TLR4, MD2, and CD14) transfected with the NF-κB-dependent reporter plasmid (293-TLR4/MD2-CD14/NF-κB) was stimulated with HSC70, and luciferase assays were performed. As shown in Figure 1A, both HSC70 and HSP70 (control) induced promoter activation in a dose-dependent manner, whereas BSA did not. Promoter activity levels with heated HSC70 and HSP70 were similar to those in unstimulated cells. When 293-TLR4/MD2-CD14/NF-κB cells were stimulated with the heated or unheated HSC70 ATPase fragments (HSC70 with deletions of the substrate-binding and lid domains), the promoter activation was at a lower level (Figure 1B). These results suggest that HSC70 as well as *E. coli* LPS induces NF-κB activation through TLR4/MD2/CD14 system.

HSP70 also induces NF-κB activation in stable HEK293-TLR2 cells transiently expressing CD14, but not in HEK293-TLR2 without CD14 [25]. Therefore, we examined NF-κB activation by HSC70 in the stable cell line 293-TLR2/CD14 (HEK293 cells constitutively expressing TLR2 and CD14) transfected with the NF-κB-dependent reporter plasmid (293-TLR2/CD14/NF-κB). As shown in Figure 1C, HSC70 as well as HSP70 (control), but not BSA, induced the promoter activation through TLR2/CD14 in a dose-dependent manner. Heated and unheated *P. gingivalis* LPSs induced promoter activation, whereas heated HSC70 and heated HSP70 reduced activation compared with the respective unheated HSPs. When the cells were stimulated with heated and unheated HSC70 ATPase fragments, only low levels of the promoter activation were observed (Figure 1D). These results suggest that both HSC70 and *P. gingivalis* LPS induce NF-κB activation through TLR2/CD14.

### Inhibitory effects of histatin 3 on HSC70-induced NF-κB activation through TLR2 and TLR4

**Figure 2:**
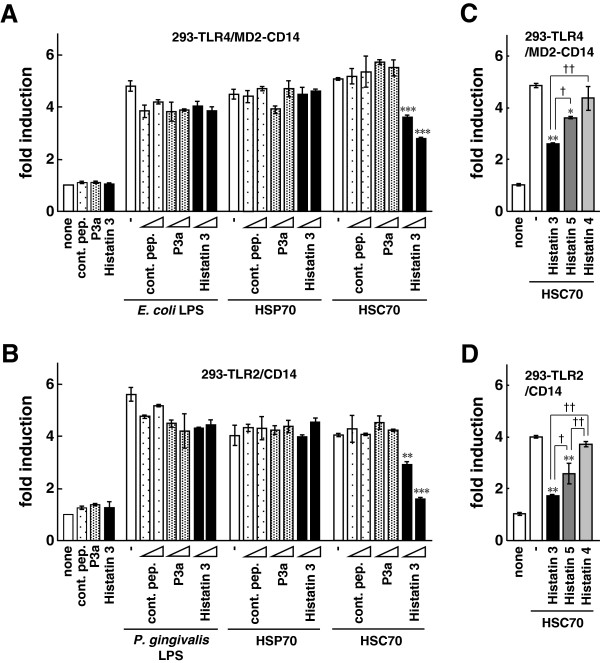
**The effect of histatin 3 on NF-κB-dependent activation by HSC70 through TLR2 and TLR4. (A, B)** Luciferase assays for NF-κB-dependent activation by HSC70 through TLR2 and TLR4 in the presence of histatin 3. 293-TLR4/MD2-CD14 **(A)** and 293-TLR2/CD14 **(B)** cells were transfected with pIgκB-Luc, and were stimulated with mixtures of the indicated peptides and LPS, HSP70, or HSC70 for 6 h. Cell lysates prepared from the stimulated cells were analyzed by luciferase assays. The values are shown as fold induction of the standardized luciferase activity over the unstimulated control (none). **, P < 0.01; ***, P < 0.001 versus stimulation with HSC70 alone. cont. pep., control peptide. **(C, D)** Luciferase assays for NF-κB-dependent activation by HSC70 through TLR2 and TLR4 in the presence of histatin family members. The procedures were same as in (A and B), except that stimulations were with mixtures of HSC70 and histatins 3, 4, or 5. Bars represent the means and range of duplicate samples. *, P < 0.05; **, P < 0.01 versus stimulation with HSC70 alone. †, P < 0.05; ††, P < 0.01.

We have previously reported that salivary protein histatin 3 binds to HSC70, but not HSP70 [7]. Here, we examined the effects of histatin 3 on NF-κB activation by HSC70 through TLR2 and TLR4. 293-TLR4/MD2-CD14/NF-κB and 293-TLR2/CD14/NF-κB cells were stimulated with mixtures of HSC70 and a control peptide, P3a (a peptide derived from the clathrin light chain that stimulates ATP hydrolysis through HSC70 [39]), or histatin 3, and luciferase assays were performed. The results showed that NF-κB-dependent promoter activation, stimulated by HSC70 through TLR4/MD2/CD14 or TLR2/CD14, was significantly suppressed by histatin 3 in a dose-dependent manner. Figure 2A shows results for TLR4/MD2/CD14 (histatin 3, 3 μM (P < 0.001) and 30 μM (P < 0.001)), and Figure 2B for TLR2/CD14 (histatin 3, 3 μM (P < 0.01) and 30 μM (P < 0.001)). However, histatin 3 did not affect promoter activation caused by LPS (from *E. coli* or *P. gingivalis*) or HSP70, in either cell type. Neither the control peptide nor P3a inhibited the LPS-, HSP70-, or HSC70-induced activation. We then tested whether other members of the histatin family were capable of suppressing NF-κB-dependent activation through TLR4/MD2/CD14 or TLR2/CD14. 293-TLR4/MD2-CD14/NF-κB and 293-TLR2/CD14/NF-κB cells were stimulated with mixtures of HSC70 and histatins 3, 4, or 5, and luciferase assays were performed. As shown in Figures 2C and 2D, HSC70-induced promoter activation was significantly reduced in the presence of histatin 3 compared with that in the presence of histatin 5 (P < 0.05 in both cell types). The suppressive effects of histatin 4 on the HSC70-mediated promoter activation were at low levels. These results suggest that histatin 3 bound to HSC70 inhibits HSC70-induced NF-κB activation both in TLR4/MD2/CD14 and TLR2/CD14 mode.

### Effect of DSG on HSC70-induced NF-κB activation through TLR2 and TLR4 in the presence of histatin 3

**Figure 3:**
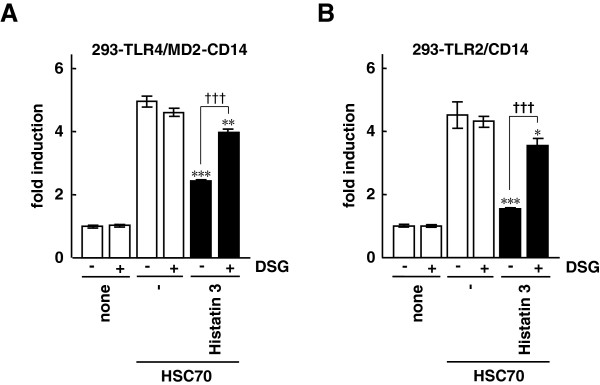
**The effect of DSG on NF-κB-dependent activation by HSC70 through TLR2 and TLR4 in the presence of histatin 3.** One day after transfection with pIgκB-Luc in 293-TLR4/MD2-CD14 **(A)** and 293-TLR2/CD14 **(B)** cells, the cells were stimulated with HSC70 with or without histatin 3 in the presence or absence of DSG for 6 h. Cell lysates prepared from the stimulated cells were analyzed by luciferase assays. The values are shown as fold induction of the standardized luciferase activity over the unstimulated control (none, DSG (-)). Bars represent the means and range of duplicate samples. *, P < 0.05; **, P < 0.01, ***, P < 0.001 versus stimulation with HSC70 alone. †††, P < 0.001.

We investigated whether the specific interaction of histatin 3 and HSC70 was necessary for inhibition of HSC70-induced NF-κB activation. The NF-κB-driven luciferase assays were performed on 293-TLR4/MD2-CD14/NF-κB and 293-TLR2/CD14/NF-κB cells treated with HSC70 along with or without histatin 3 in the presence or absence of DSG. DSG is a synthetic analog of spergualin, a natural product from *Bacillus laterosporus*, and has a peptidomimeric structure. It binds particularly well to HSC70 and is considered to preclude peptide binding to HSC70 [40,41]. The results demonstrated that in the presence of histatin 3 and DSG HSC70-stimulated NF-κB-dependent promoter activity was significantly higher than with histatin 3 alone in both 293-TLR4/MD2-CD14 (Figure 3A, P < 0.001) and 293-TLR2/CD14 (Figure 3B, P < 0.001) cells. These results suggest that the specific binding of histatin 3 to HSC70 is important for inhibition of HSC70-mediated activation of TLR2 and TLR4 signaling activation.

### Production of inflammatory cytokines by HSC70 stimulation in HGFs

**Figure 4:**
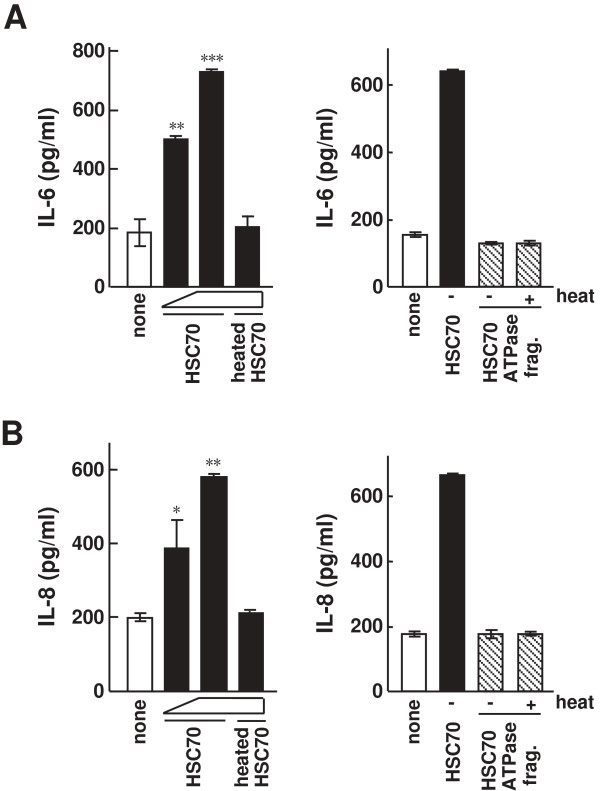
**The effect of HSC70 on inflammatory cytokine production in HGFs. (A, B)** IL-6 and IL-8 production by HSC70 stimulation in HGFs. Unheated HSC70 (7 and 70 nM) and heated HSC70 (70 nM) were added to HGFs (left). Unheated or heated HSC70 ATPase fragment (70 nM each) were also added to HGFs (right). The cells were cultured for 24 h. The amounts of IL-6 **(A)** and IL-8 **(B)** in the culture media were measured by ELISAs. Bars represent the means and range of duplicate samples. *, P < 0.05; **, P < 0.01; ***, P < 0.001 versus unstimulated (none).

It is unknown whether HSC70 is involved in inflammatory response in HGFs, especially in the production of inflammatory cytokines. To examine this possibility, HGFs were stimulated with HSC70 for 24 h, and the amounts of IL-6 and IL-8 in the culture media were measured by ELISAs. As shown in Figures 4A and 4B, unheated HSC70 significantly induced cytokine production in HGFs in a dose-dependent manner, whereas heated HSC70 did not induce cytokine production (HSC70, 7 nM (P < 0.01) and 70 nM (P < 0.001) in Figure 4A; 7 nM (P < 0.05) and 70 nM (P < 0.01) in Figure 4B). When HGFs were stimulated with the unheated and heated HSC70 ATPase fragments, the levels of IL-6 and IL-8 were similar to those in unstimulated cells. These results suggest that HSC70 induces inflammatory cytokine production in HGFs.

### Inhibitory effect of anti-TLR antibodies on inflammatory cytokine production stimulated by HSC70 in HGFs

**Figure 5:**
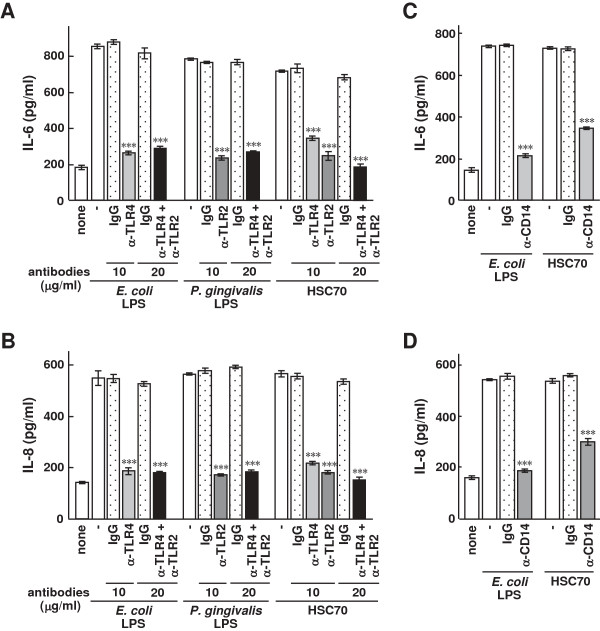
**The effect of anti-TLR antibodies on HSC70-stimulated inflammatory cytokine production in HGFs. (A)** IL-6 and **(B)** IL-8 production with LPS or HSC70 in the presence of anti-TLR2 and anti-TLR4 antibodies in HGFs. HGFs were cultured for 1 h after the addition of the antibodies and were then stimulated with LPSs or HSC70 for 24 h, and ELISAs for IL-6 and IL-8 were performed on the cultured media. **(C)** LPS- and HSC70-stimulated IL-6 and **(D)** IL-8 production in the presence of anti-CD14 antibody in HGFs. Assays were performed as described in the “Methods.” IgG served as the antibody control. Bars represent the means and range of duplicate samples. ***, P < 0.001 versus stimulation with LPS or HSC70 alone.

To examine whether inflammatory cytokine production stimulated by HSC70 was dependent on TLR2 or TLR4 in HGFs, we performed experiments using anti-TLR2 and anti-TLR4 antibodies. As shown in Figure 5A, levels of HSC70-stimulated IL-6 production in HGFs with anti-TLR4, anti-TLR2, and both antibodies significantly decreased (P < 0.001). LPS-stimulated IL-6 production also significantly decreased in the presence of anti-TLR4 and both anti-TLR2 and anti-TLR4 antibodies (P < 0.001). The patterns of HSC70- and LPS-stimulated IL-8 production in the presence of the antibodies were similar to those of IL-6 production under the same condition (Figure 5B). Next, we examined the effect of anti-CD14 antibody on HSC70- and LPS-stimulated inflammatory cytokine production in HGFs. HSC70-stimulated IL-6 and IL-8 production significantly decreased in the presence of this antibody (P < 0.001) (Figures 5C and 5D). The antibody also caused a decrease in cytokine production in LPS-stimulated HGFs. The levels of IL-6 and IL-8 production under HSC70 stimulation were approximately1.6-fold higher than those by LPS stimulation. This behavior is similar to the previously reported findings for HSP70 stimulation of cytokine production through a CD14-dependent pathway [37]. These results suggest that HSC70-stimulated inflammatory cytokine production in HGFs is dependent on TLR2, TLR4, and CD14.

### Inhibitory effect of histatin 3 on HSC70-stimulated inflammatory cytokine production in HGFs

**Figure 6:**
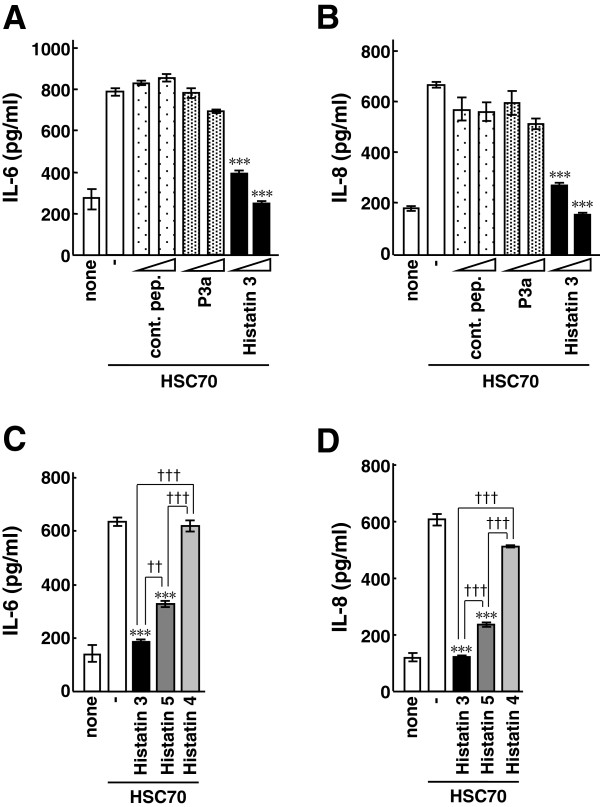
**The effect of histatin 3 on HSC70-stimulated inflammatory cytokine production in HGFs. (A)** HSC70-stimulated IL-6 and **(B)** IL-8 production in the presence of histatin 3 in HGFs. HGFs were stimulated with HSC70 in the presence of control peptide (cont. pep.), P3a, or histatin 3 for 24 h, and the amounts of IL-6 and IL-8 in the culture media were measured by ELISAs. Bars represent the means and range of duplicate samples. ***, P < 0.001 versus stimulation with HSC70 alone. **(C)** IL-6 and **(D)** IL-8 production in HGFs after stimulation with HSC70 in the presence of histatin family members. HGFs were stimulated with HSC70 in the presence of histatins 3, 4, or 5 for 24 h, and the amounts of IL-6 and IL-8 in the culture media were measured by ELISAs. Bars represent the means and range of duplicate samples. ***, P < 0.001 versus stimulation with HSC70 alone. ††, P < 0.01; †††, P < 0.001.

Histatin 3 was found to inhibit NF-κB activation through TLR2 and TLR4 (Figure 2). We then examined the effect of histatin 3 on HSC70-stimulated inflammatory cytokine production in HGFs. HSC70 and histatin 3 were added to HGFs, and the amounts of IL-6 and IL-8 released into the culture media were measured by ELISAs. As shown in Figures 6A and 6B, HSC70-stimulated cytokine production significantly decreased in the presence of histatin 3, in a dose-dependent manner (histatin 3, 0.15 μM (P < 0.001) and 1.5 μM (P < 0.001)). The control peptide and P3a did not appreciably affect the cytokine production. We then investigated whether other members of the histatin family were capable of inhibiting HSC70-stimulated cytokine production in HGFs. Histatins 3, 4, and 5 were mixed with HSC70 and used to stimulate HGFs, and the amounts of IL-6 and IL-8 in the culture media were measured. As shown in Figures 6C and 6D, the inhibitory effect of histatin 3 on the cytokine production was significantly higher than that of histatin 5 (IL-6, P < 0.01; IL-8, P < 0.001). These results suggest that histatin 3 is an inhibitory factor of HSC70-stimulated inflammatory cytokine production in HGFs.

### Effects of histatin 3 on HSC70-stimulated MAPK phosphorylation and IκB-α degradation in HGFs

**Figure 7:**
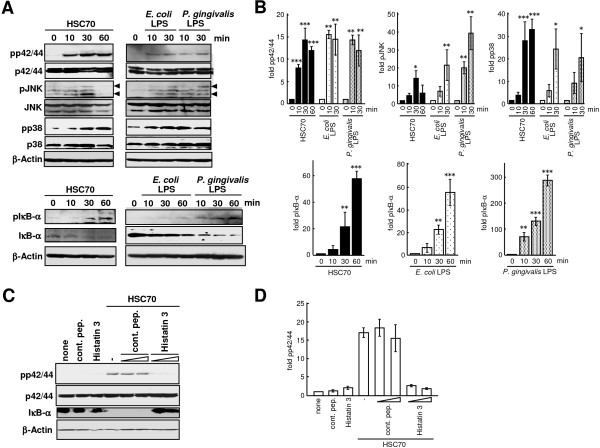
**MAPK phosphorylation and IκB-α degradation induced by HSC70 and the effect of histatin 3. (A)** MAPK and IκB-α phosphorylation and IκB-α degradation after HSC70 or LPS stimulation. HGFs were stimulated with LPSs or HSC70 for the indicated time periods. Phosphorylation of p42/44 (ERK), JNK, p38, and IκB-α (pp42/44, pJNK, pp38, and pIκB-α) and degradation of IκB-α were examined by Western blotting with the respective antibodies. **(B)** Densitometry analysis of Western blotting from **(A)**. Phosphorylation of p42/44, JNK, p38, and IκB-α was quantified as levels relative to respective MAPKs and IκB-α after normalization with β-actin. Bars represent the means of 3 experiments; error bars show standard deviations. The values are shown as fold induction of phosphorylation over the “0 min” sample. *, P < 0.05; **, p < 0.01; ***, p < 0.001. **(C)** Histatin 3 inhibition of HSC70-stimulated p42/44 phosphorylation and IκB-α degradation. HGFs were stimulated with HSC70 in the presence of control peptide (cont. pep.) or histatin3 for 30 min. The effects of histatin 3 on p42/44 phosphorylation and IκB-α degradation were examined by Western blotting with the indicated antibodies. **(D)** Phosphorylation of p42/44 was quantified as levels relative to p42/44 after normalization with β-actin. Bars represent the means of 3 experiments; error bars show standard deviations. The values are shown as fold induction of phosphorylation over the “0 min” sample.

We next examined whether HSC70 stimulated MAPKs (extracellular signal-regulated kinases (ERK (p42/44)), JNK, and p38) phosphorylation and IκB-α degradation in HGFs. HGFs were cultured with HSC70 for 10, 30, and 60 min, and extracted proteins were analyzed by Western blotting with anti-MAPK and anti-IκB-α antibodies. As shown in Figures 7A and 7B, enhanced phosphorylation of p42/44, JNK, p38, and IκB-α and stimulated significant degradation of IκB-α by HSC70 were observed. LPSs also stimulated significantly both the phosphorylation of MAPKs and IκB-α and the degradation of IκB-α.

To examine whether HSC70-induced ERK phosphorylation and IκB-α degradation were inhibited by histatin 3, HGFs were stimulated with HSC70 in the presence of histatin 3, and Western blotting was performed. As shown in Figures 7C and 7D, p42/44 phosphorylation induced by HSC70 in the presence of histatin 3 was lower than that in the absence of histatin 3 or in the presence of the control peptide. Stimulation of either the control peptide or histatin 3 alone decreased levels of p42/44 phosphorylation. NF-κB activation by stimulation with the control peptide or histatin 3 alone was also diminished in 293-TLR4/MD2-CD14 and 293-TLR2/CD14 cells (Figures 2A and 2B). HSC70 stimulated IκB-α degradation in the presence of the control peptide, but not in the presence of histatin 3. These results suggest that histatin 3 inhibits HSC70-mediated MAPK phosphorylation and IκB-α degradation in HGFs.

### Histatin 3 binding-induced protease-resistant conformation of HSC70

**Figure 8:**
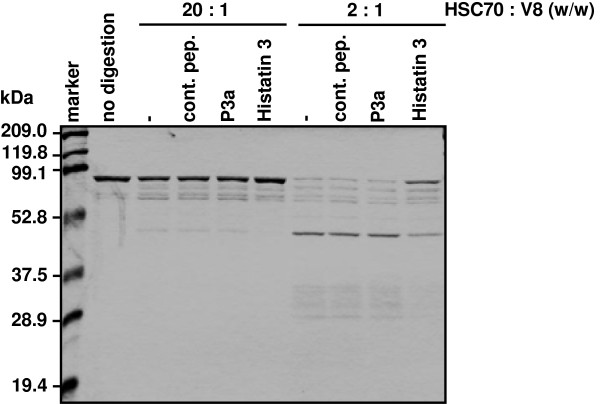
**Protease-resistant conformation in histatin 3-bound HSC70.** HSC70 was mixed with control peptide (cont. pep.), P3a, or histatin 3, and the mixtures were analyzed by limited V8 protease proteolysis. The ratios of V8 protease to HSC70 were 2:1 and 20:1 (w/w). The mixtures were incubated at 30°C for 1.5 h, and the proteolytic fragments were separated on 12% SDS-PAGE gels and stained with Coomassie Brilliant Blue.

Histatin 3 prevented HSC70-stimulated NF-κB activation through TLR2 and TLR4 (Figure 2) and inflammatory cytokine production in HGFs (Figure 6). Therefore, we assumed that histatin 3 binding to HSC70 had some effect on the conformation of HSC70. To examine this assumption, HSC70 was mixed with histatin 3, and the mixture was analyzed by limited V8 protease proteolysis. As shown in Figure 8, HSC70 with histatin 3 was relatively more resistant to digestion by V8 protease with HSC70 with the control peptide or P3a. Histatin 3 did not inhibit the activity of V8 protease (data not shown). These results suggest that histatin 3 may affect the conformation of HSC70 upon binding.

## Discussion

It has been reported that extracellular HSP70 induces inflammatory cytokine production through TLR2 and TLR4 pathway in human monocytes [25]. However, it is not known whether HSC70 activates TLR2 and TLR4 signaling in oral cells, and if so, whether intravital (bioactive) factors that inhibit HSC70 function exist in saliva. In this study, we found that HSC70, along with MD2/CD14, stimulated TLR4 and that HSC70, along with CD14, stimulated TLR2, resulting in the induction of NF-κB-dependent activation. HSC70 also induced inflammatory cytokine production, MAPK phosphorylation, and IκB-α degradation in HGFs. Histatin 3 inhibited those effects of HSC70. Moreover, we believe that histatin 3 may affect conformation of HSC70 upon binding, presumably inhibiting HSC70 function.

In experiments related to TLR stimulation, stimulating reagents might be contaminated with endotoxin. Our studies showed that boiling (95°C, 20 min) abrogated the effects of HSC70 induction, but not of LPS induction (Figure 1A). Moreover, polymyxin B, an LPS antagonist, abrogated the effects of LPS induction, but not of HSC70 induction (data not shown). In addition, endotoxin activity in the HSC70 reagents was analyzed by the limulus amebocyte lysate (LAL) assay and was found to be low. A recent study has revealed that TLR4 activation is induced by the HSP70 reagents which may include a small amount of endotoxin. The results of the study suggest that the stimulatory effect depends on HSP70, even in the presence of a small amount of endotoxin, and the structural integrity of HSP70 is essential [42]. Our results showed that histatin 3 significantly inhibits HSC70-stimulated NF-κB activation and inflammatory cytokine production, despite a slight contamination of HSC70 reagents with endotoxins (Figures 2A and 6). Consequently, we can conclude that HSC70 provably affects NF-κB activation and inflammatory cytokine production and histatin 3 may inhibit those effects upon its binding to HSC70. It is also possible that reagents used in stimulating experiments were contaminated with lipoproteins. We observed a decrease in the levels of NF-κB activation after stimulation with heated HSC70 (Figure 1C). Furthermore, the levels of inflammatory cytokine production after treatment with heated HSC70 were very low (Figure 4). In addition, histatin 3 significantly inhibited NF-κB activation and inflammatory cytokine production caused by HSC70 stimulation (Figures 2B and 6), although HSPs possess an affinity for lipids [43,44]. It is also difficult to precisely quantify the small amount of HSC70 associated with lipids in the HSC70 reagents. Consequently, if the HSC70 reagents contain HSC70 associated with lipids (even to a very small extent), our results might reflect the function of HSC70 in various physiological forms (for example, HSC70 that exists under the various physiological conditions of the oral cavity), because HSC70 derived from the cells might form lipoproteins [42,43]. We can conclude that histatin 3 binding to HSC70 may inhibit HSC70 activity.

A previous study has reported that HSC70 is released from injured cells [45]. The release of HSC70 from glial and K562 erythroleukemic cells has been also observed [46,47]. Our findings show that extracellular HSC70 stimulates TLR2 and TLR4 and increases the production of inflammatory cytokines in HGFs (Figure 4). Therefore, we suggest that HSC70 as well as other HSPs (e.g., HSP70 and HSP60) may function as a DAMP for TLRs and elicit inflammatory responses. The release of HSC70 has also been observed in the heart, contributing to the postischemic myocardial inflammatory response and to cardiac dysfunction [48]. Inflammatory response in the oral cavity is also observed in oral diseases and injuries. It is tempting to speculate that HSC70 released from the damaged cells may stimulate oral cells such as HGFs. Our present findings suggest that inflammatory cytokine production stimulated by the released HSC70 might be inhibited by histatin 3 in saliva in HGFs, histatin 3 may be involved in inflammatory processes in the oral cavity.

Our previous study demonstrated the HSC70-binding ability of histatins [7]. The study showed that histatin 3 bound to the substrate-binding domain of HSC70 more strongly than histatin 5 and that histatin 4 did not bind to HSC70. Our present findings indicate that the inhibitory effects of histatin 5 on HSC70-stimulated NF-κB-dependent activation and inflammatory cytokine production significantly reduced compared with those of histatin 3 (Figures 2C, 2D, 6C, and 6D). Consequently, the strength of the association between various histatins and HSC70 may be related to the function of the complex.

In addition, it seems very likely that the primary structure of HSC70 is necessary for the function of HSC70 in TLR-mediated processes. Our findings showed that full-length HSC70 and not the HSC70 ATPase fragment, can stimulate TLRs (Figures 1 and 4). In fact, a previous study reported that the substrate-binding domain of HSC70 is required to induce the myocardial inflammatory response [48]. In addition, conformation of HSC70 is also important for its correct functioning. Our findings show that a relatively protease-resistant conformation is formed upon histatin 3 binding to HSC70, but not in the presence of the control peptide, P3a (Figure 8), or DSG (data not shown). These results imply the possibility that there are some effects on conformation of HSC70. In fact, previous studies have reported that the peptide-binding domain of HSC70, as well as the ATPase domain of DnaK (the *E. coli* homolog of HSC70) is capable of undergoing conformational changes [49-51]. Thus, both the primary structure and other conformations of HSC70 may contribute to the activation of TLR signaling.

The innate host defense system recognizes foreign substances and tries to decrease their effects. One of the various host defense factors, pulmonary surfactant protein A downregulates the activation of TLR2 signaling by PGN [52]. An inhibitory peptide of TLR signaling, P13, inhibits both *in vitro* and *in vivo* LPS-induced inflammatory responses [53]. However, the precise mechanisms of this action have not been clarified. Histatin 3 is a peptide that binds directly to HSC70 and inhibits HSC70-induced TLR2 and TLR4 cell signaling (Figures 2, 6, and 7). Therefore, the results presented here provided the first evidence that histatin 3 is a salivary bioactive molecule. This molecule may prevent early-stage TLR signaling activation by interacting with TLR stimulators (ligands), such as HSC70, a putative ligand found in the oral cells.

## Conclusion

In this study, we demonstrated the production of inflammatory cytokines in oral cells constitutively expressing HSP and the inhibition of this production by a salivary protein. Thus, this salivary protein has anti-inflammatory activity as well as already reported antimicrobial activity. It is possible that this salivary protein may resolve the HSP-mediated inflammatory response in the oral cavity. The present findings further our understanding of the functions and mechanisms of actions of salivary proteins.

## Abbreviations

DMEM: Dulbecco’s modified Eagle medium; DSG: 15-deoxyspergualin; ELISA: Enzyme-linked immunosorbent assay; ERK: Extracellular signal-regulated kinases; EU: Endotoxin units; FBS: Fetal bovine serum; HGFs: Human gingival fibroblasts; HSC70: Heat shock cognate protein 70; HSP: Heat shock protein; IL: Interleukin; JNK: JUN-N-terminal protein kinase; LAL: Limulus amebocyte lysate; LPS: Lipopolysaccharide; MAPK: Mitogen-activated protein kinase; NF-κB: Nuclear factor-κB; PGN: Peptidoglycan; TLR: Toll-like receptor.

## Competing interests

The authors declare that they have no competing interests.

## Authors’ contributions

YI designed, conducted, analyzed and interpreted the data, and prepared the figures and manuscript. PLW designed and interpreted data, and helped prepare the manuscript. Both authors read and approved the final manuscript.
